# Occurrence and Determinants of Postpartum Maternal Morbidities and Disabilities among Women in Matlab, Bangladesh

**DOI:** 10.3329/jhpn.v30i2.11308

**Published:** 2012-06

**Authors:** J. Ferdous, A. Ahmed, S.K. Dasgupta, M. Jahan, F.A. Huda, C. Ronsmans, M. Koblinsky, M.E. Chowdhury

**Affiliations:** ^1^icddr,b, GPO Box 128, Dhaka 1000, Bangladesh; ^2^London School of Hygiene & Tropical Medicine, London, UK; ^3^John Snow Inc., Arlington, Virginia, USA

**Keywords:** Complicated births, Disabilities, Long-term morbidities, Normal uncomplicated births, Obstetric complications, Perinatal death, Postpartum morbidities, Bangladesh

## Abstract

The burden of maternal ill-health includes not only the levels of maternal mortality and complications during pregnancy and around the time of delivery but also extends to the standard postpartum period of 42 days with consequences of obstetric complications and poor management at delivery. There is a dearth of reliable data on these postpartum maternal morbidities and disabilities in developing countries, and more research is warranted to investigate these and further strengthen the existing safe motherhood programmes to respond to these conditions. This study aims at identifying the consequences of pregnancy and delivery in the postpartum period, their association with acute obstetric complications, the sociodemographic characteristics of women, mode and place of delivery, nutritional status of the mother, and outcomes of birth. From among women who delivered between 2007 and 2008 in the icddr,b service area in Matlab, we prospectively recruited all women identified with complicated births (n=295); a perinatal mortality (n=182); and caesarean-section delivery without any maternal indication (n=147). A random sample of 538 women with uncomplicated births, who delivered at home or in a facility, was taken as the control. All subjects were clinically examined at 6-9 weeks for postpartum morbidities and disabilities. Postpartum women who had suffered obstetric complications during birth and delivered in a hospital were more likely to suffer from hypertension [adjusted odds ratio (AOR)=3.44; 95% confidence interval (CI)=1.14-10.36], haemorrhoids (AOR=1.73; 95% CI=1.11-3.09), and moderate to severe anaemia (AOR=7.11; 95% CI=2.03-4.88) than women with uncomplicated normal deliveries. Yet, women who had complicated births were less likely to have perineal tears (AOR=0.05; 95% CI=0.02-0.14) and genital prolapse (AOR=0.22; 95% CI=0.06-0.76) than those with uncomplicated normal deliveries. Genital infections were more common amongst women experiencing a perinatal death than those with uncomplicated normal births (AOR=1.92; 95% CI=1.18-3.14). Perineal tears were significantly higher (AOR=3.53; 95% CI=2.32-5.37) among those who had delivery at home than those giving birth in a hospital. Any woman may suffer a postpartum morbidity or disability. The increased likelihood of having hypertension, haemorrhoids, or anaemia among women with obstetric complications at birth needs specific intervention. A higher quality of maternal healthcare services generally might alleviate the suffering from perineal tears and prolapse amongst those with a normal uncomplicated delivery.

## INTRODUCTION

The status of maternal health in poor countries is often described in terms of maternal mortality alone, despite the evidence that far more women suffer from morbidities/disabilities relating to pregnancy and childbirth (1). Every year, over 300,000 women die due to complications in pregnancy and childbirth, and approximately 15% of all pregnant women or about 20 million women suffer from acute severe obstetric complications, including haemorrhage, obstructed/prolonged labour, pre-eclampsia/eclampsia, puerperal sepsis, and septic abortion (2-4). The burden of maternal ill-health extends beyond these complications and includes different short- and long-term morbid conditions that can result from acute obstetric complications or poor management at delivery (5,6).

The postpartum morbid consequences include problems, such as postpartum infection, anaemia, perineal tears, urinary tract infection, and depression; others defined in the literature as long-term morbidities/disabilities include incontinence, fistula, pelvic inflammatory disease, genital prolapse, hypertension, haemorrhoids, nerve damage, pituitary failure, anaemia, and infertility. Many of these maternal morbidities and disabilities may arise during delivery or in the first 1-2 week(s) following delivery and can become chronic if not cared for appropriately. Globally, 15-20 million women each year are estimated to suffer from these postpartum and long- term morbidities/disabilities (7).

In Bangladesh, a few studies have reported the consequences of acute maternal morbidities. Fortney *et al*. reported 28.6% of women in Bangladesh suffering from long-term pregnancy-related morbidities (8). A 1996 study estimated that 9 million Bangladeshi ever-married women had long-lasting obstetric morbidities/disabilities, such as fistula (2%), uterine prolapse (15%), incontinence (5%), and haemorrhoids (4%) (9). A community survey in a Dhaka slum reported that more than three-quarters of women suffered from a non-trivial illness during the first 6 weeks postpartum (10). All these studies are based on self-reported morbidities. Though women's perceptions of their own ill-health are important, these are not necessarily clinically valid or reliable (11-13). A more robust measurement of the consequences of acute maternal morbidities and intrapartum care based on clinical data is needed for programme planning.

This study aims at filling the information gap by investigating the association of postpartum maternal morbidities/disabilities with various acute obstetric complications arising during pregnancy or delivery, and with sociodemographics and other key characteristics of women at delivery.

## MATERIALS AND METHODS

### Study design and settings

A prospective cohort study was conducted among women who delivered during 2007-2008 in the icddr,b field site in Matlab, located about 55 km southeast of Dhaka. Matlab is a rural area in Bangladesh where the main occupations of people are fishing and agriculture. In this setting, women's literacy rate is similar to that in other rural areas of Bangladesh (50.6%) (14). Since 1966, a health and demographic surveillance system has been systematically collecting vital statistics on a population of about 220,000 in the icddr,b's field site in Matlab.

Since 1987, a safe motherhood intervention programme has been in place covering about 110,000 of the population—essentially all those living in the icddr,b service area. Pregnancy, delivery and postpartum services are provided by midwives at four health subcentres and by female medical doctors at the Matlab Hospital of icddr,b providing basic emergency obstetric care. Women may also use any one of the other 30 delivery facilities in the nearby area. Given the ability to track all women with deliveries in the study site, this setting provides a unique opportunity to identify women with postpartum morbidities or with long-term consequences of acute maternal complications or normal deliveries and their determinants. The use of facilities for delivery was relatively high (63%) in this area during the study period compared to other parts of Bangladesh (23%) (15,16).

### Study population and selection of subjects

The study subjects (n=1,162 initially) were selected from those Matlab women who delivered between 2007 and 2008 to represent four categories of deliveries–those with a uncomplicated normal vaginal birth, those who suffered a perinatal death, those who had severe or less-severe complications during pregnancy or delivery, or those who had a caesarean section but no recorded maternal indication (Fig.). All women diagnosed with a severe acute maternal complication and half of those with a less-severe complication (n=295) were included in the sample. They had been diagnosed through a review of facility-records (n=1,927) of all women who delivered in the Matlab Hospital of icddr,b or those attending a public or private hospital with comprehensive emergency obstetric care (CEmOC) in Matlab or the nearby Chandpur district town (16). All women with a perinatal death (n=182) occurring either at home or a health facility during the study period were included; another group comprised all women with a caesarean section without any maternal indication (n=147). A random sample of women with normal vaginal deliveries without any complications (n=538) were included in the study for comparison.

**Fig.**
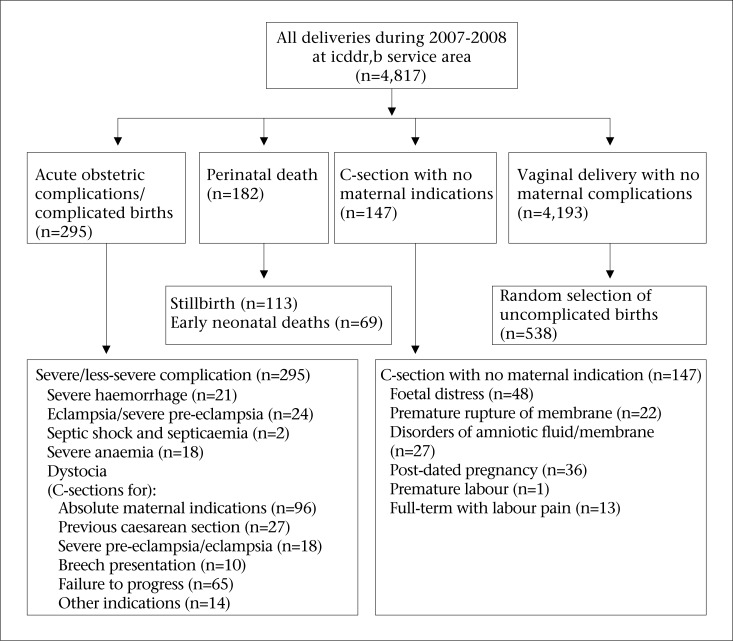
Selection of subjects by category of delivery

An uncomplicated birth is the outcome of a normal vaginal delivery either at home or facility. An uncomplicated birth at home was defined as normal vaginal delivery of a singleton, cephalic and full-term (>37 weeks of gestation) baby with neither a perinatal death nor hospitalization for postpartum complications up to 2 weeks of termination of pregnancy. An uncomplicated birth at facility was defined as a normal vaginal delivery at facility, with no recorded obstetric complications of the mother who had a singleton, cephalic and full-term pregnancy outcome, with no perinatal death.

### Procedures of physical examination

All 1,162 women initially enrolled in the study were asked to attend the nearest icddr,b subcentre at around 6-9 weeks postpartum for physical examination for postpartum morbidities. A Female Field Worker (FFW) visited each enrolled mother at home to invite her to come to a nearby icddr,b subcentre on a scheduled date for the postnatal medical check-up.

The examination was carried out by a female physician to measure height and weight and identify the following conditions: incontinence, fistula, urinary infection, genital prolapse, anaemia, hypertension, genital infection, perineal tear, breast problems, haemorrhoids, foot drop, and other morbidities/disabilities. Blood and urine samples were measured and assessed for anaemia and urinary tract infection (UTI) respectively (17,18). The abdomen was inspected for any signs of infection, including the scar condition for mothers with caesarean section. Each part of the genital tract (vulva, vagina, cervix, body of the uterus, adnexa, and pouch of Douglas) was examined. Inspection was first carried out to observe the vulval skin condition for signs of infection and discharge, perineal tear, prolapse, and haemorrhoids. Per-speculum examination was carried out to determine vaginal discharge and the condition of the cervix, and to diagnose vaginal prolapse or vesicovaginal/rectovaginal fistula. Bi-manual pelvic examination was also performed to observe the size, shape, position, mobility, consistency, and tenderness of the cervix and uterus. Mothers diagnosed with any morbidity/disability were treated following standard treatment guidelines by the study physician or referred (for all severe perineal tears and second- and third-degree uterine prolapse) to a specialized hospital for surgical intervention. Informed consent to participate in the examination and the study was taken for each woman who came for postpartum physical examination.

### Definition and tests for morbidities/disabilities

Morbidities/disabilities were measured at around 6-9 weeks after birth, using standard clinical protocols and specific tests as specified in Table 1. Operational definitions of the morbidities were used through a review of the WHO guidelines and consultations with experienced local obstetricians and researchers.

**Table 1.** Maternal morbidities/disabilities: definitions, clinical examination, and specific biomedical tests

Maternal morbidity/disability	Definition	Clinical examination and specific biomedical tests
Anaemia: WHO Guideline (2003)	Severe anaemia—Haemoglobin level <7 g/dL Moderate anaemia—Haemoglobin level 7-9 g/dL Mild anaemia—Haemoglobin level >9-11 g/dL (19)	HemoCue Haemoglobinometry
Breast problems	Breast infection/Mastitis—pain, localized mass, tenderness, redness of breast Abscess—Breast mass with fluctuance (20)	Visual inspection and manual palpation of the breast
Foot drop	Weakness on dorsiflexion of the foot with paraesthesia in the foot and second toes. A difficulty in turning the foot upwards (21)	Clinical examination
Hypertension	Systolic blood pressure ≥140 mmHg or diastolic blood pressure ≥90 mmHg (19)	Sphygmomanometry
External haemorrhoids	Varicosities of the veins that occur outside the anal verge (the distal end of the anal canal), sometimes extruded as thrombosed structure strangulated by anal sphincter (20)	Clinical examination: Women were placed in a left lateral position to inspect anus and perineum. Visible bleeding or swollen/thrombosed varicose vein was diagnosed as external haemorrhoids
Urinary incontinence	Involuntary loss of urine weekly or more often measured by Cough Reflex Test (21)	Cough Reflex Test: Women were asked to take 500 mL of water 45 minutes prior to pelvic examination and advised to walk around. During visual inspection, women were asked to cough strongly to see if urine was released
Perineal tear	Injury to the perineum involving perineal muscles with or without anal sphincter involvement	Clinical examination
	First-degree perineal tear: Injury to the vaginal mucosa and connective tissue	
	Second-degree perineal tear: Injury to the perineum involving vaginal mucosa and perineal muscles without involving anal sphincter	
	Third-degree perineal tear: Injury to the perineum involving anal sphincter complex	
	Fourth-degree perineal tear: Injury to the perineum involving anal sphincter complex and rectal mucosa (19)	
Prolapse	Uterine prolapse: Uterus descending into vaginal canal with cervix as leading edge	Clinical examination
	Uterovaginal prolapse: Uterine prolapse pulling down the vagina	
	Vaginal prolapse: One or more region of vaginal wall protrude into the vaginal canal—(i) cystocele involves upper anterior vaginal wall; (ii) urethrocele: lower anterior vaginal wall; (iii) rectocele involves lower posterior vaginal wall; (iv) enterocele: upper posterior vaginal wall (22)	
Pelvic inflammatory disease (PID)	Bilateral lower abdominal and pelvic pain, dull in nature, fever (temperature >38 °C), lassitude, headache, abnormal vaginal discharge which becomes purulent and/or copious, nausea, vomiting, and dyspareunia (difficult and/or painful coital act)	Clinical examination
Or, fever and lower abdominal pain (without missed period, recent delivery/abortion, abdominal guarding, rebound tenderness, abnormal vaginal bleeding, abdominal mass), with lower abdominal tenderness or cervical motion tenderness and vaginal discharge (21)	
Obstetric fistula	An abnormal opening between the vagina and the bladder or rectum with involuntary escape of urine (VVF)/flatus and/or faeces (RVF) (22)	Clinical examination/per-speculum examination
Urinary tract infection (UTI)	Burning urination (WHO, 2003) (19)	Urinary infections were assessed with Oxidase Strip Test (18)

### Statistical analysis

Pregnancy-records of all selected women were linked with their records from the icddr,b Health and Demographic Surveillance System (HDSS) to determine the socioeconomic characteristics of the women and their households. Asset quintiles were generated using principal component analysis based on possession of household commodities/utilities, construction materials of houses, and ownership of land and cattle. Descriptive analysis was used for reporting occurrence of the postpartum morbidities/disabilities. Pearson's chi-square test was used for the crude association between postpartum morbidities/disabilities and selected reproductive and sociodemographic characteristics of the women. By using logistic regression, risk factors for postpartum morbidities/disabilities adjusted for potential confounders were identified. Stata (version 10) statistical software package was used for analysis.

## RESULTS

Among the 1,162 enrolled mothers, 1,037 (89.2%) came to health centres and provided consent for a physical examination; 54 (4.7 %) were lost to follow-up, and 71 (6.1%) refused to participate. The mean number of days elapsed between delivery and physical examination was 66 days (range: 42-145) or roughly 9 weeks. In the analysis, women who experienced both a perinatal death and an acute obstetric complication were excluded from the latter category to avoid double counting. Thus, at the time of examination, the categories of women included: 274 who had experienced a severe or less-severe obstetric complication (excluding 21 with a perinatal death), 156 with a perinatal mortality, 125 with a caesarean section without maternal indication, and 482 with uncomplicated birth. The sociodemographic and reproductive characteristics of the examined mothers are shown in Table 2.

**Table 2.** Reproductive and sociodemographic characteristics of women examined in the study by type of delivery

Characteristics	Complicated births: severe/less-severe acute obstetric complications (n=274) No. (%)	C-section without maternal indications (n=125) No. (%)	Perinatal death (n=156) No. (%)	Normal uncomplicated births (n=482) No. (%)	Examined women (n=1,037) No. (%)
Age (years) of mothers	<20	43 (15.7)	24 (19.2)	28 (18.0)	70 (14.5)	165 (15.9)
	20-24	89 (32.5)	40 (32.0)	45 (28.9)	153 (31.7)	327 (31.5)
	25-29	75 (27.4)	37 (29.6)	42 (26.9)	134 (27.8)	288 (27.8)
	30-34	40 (14.6)	13 (10.4)	26 (16.7)	87 (18.1)	166 (16.0)
	35+	27 (9.9)	11 (8.8)	15 (9.6)	38 (7.9)	91 (8.8)
Parity	1	112 (40.9)	59 (47.2)	74 (47.4)	117 (24.3)	362 (34.9)
	2-4	119 (43.4)	46 (36.8)	54 (34.6)	296 (61.4)	515 (49.7)
	5+	10 (3.7)	2 (1.6)	3 (1.9)	21 (4.4)	36 (3.5)
	Unknown	33 (12.0)	18 (14.4)	25 (16.3)	48 (10.0)	124 (12.0)
Formal education (years of schooling)	0	25 (9.1)	10 (8.0)	24 (15.4)	77 (16.0)	136 (13.1)
	1-4	22 (8.0)	9 (7.2)	11 (7.1)	47 (9.8)	89 (8.6)
	5-7	49 (17.9)	15 (12.0)	39 (25.0)	140 (29.0)	243 (23.4)
	8+	149 (54.4)	80 (64.0)	55 (35.2)	169 (35.0)	453 (43.7)
	Unknown	29 (10.6)	11 (8.8)	27 (17.3)	49 (10.2)	116 (11.2)
Asset quintile	Poorest	23 (8.4)	12 (9.6)	24 (15.4)	96 (20.0)	155 (15.0)
	Less poor	41 (15.0)	13 (10.4)	28 (18.0)	80 (16.6)	162 (15.7)
	Middle	35 (12.8)	15 (12.0)	28 (18.0)	97 (20.1)	175 (16.9)
	Richer	48 (17.5)	28 (22.4)	23 (14.7)	86 (17.8)	185 (17.8)
	Richest	96 (45.0)	50 (40.0)	37 (23.7)	87 (18.0)	270 (26.0)
	Unknown	31 (11.3)	7 (5.6)	16 (10.3)	36 (7.5)	90 (8.7)
BMI (kg/m^2^)	<18.5	38 (13.9)	18 (14.4)	24 (15.4)	90 (18.7)	170 (16.5)
	18.5-25.0	186 (67.9)	87 (69.6)	115 (73.7)	359 (74.5)	747 (72.0)
	>25	50 (18.3)	20 (16.0)	17 (10.9)	33 (6.9)	120 (11.6)
Pregnancy outcome	Stillbirth	0 (0.0)	0 (0.0)	99 (61.6)	0 (0.0)	99 (9.5)
	Livebirth	274 (100.0)	125 (100.0)	57 (38.4)	482 (100.0)	938 (90.5)
	Early neonatal death	-	-	-	-	57 (5.5)
Mode of delivery	Vaginal delivery	50 (18.3)	0 (0.0)	138 (88.5)	482 (100.0)	670 (64.6)
	Caesarean section: total	224 (81.8)	125 (100.0)	18 (11.5)	0 (0.0)	367 (35.4)
Place of delivery	Home	5 (1.8)	0 (0.0)	45 (28.9)	250 (51.9)	300 (29.1)
	Health subcentre	0 (0.0)	0 (0.0)	13 (8.3)	99 (20.5)	112 (10.9)
	Hospital	268 (90.8)	125 (100.0)	98 (62.8)	133 (27.6)	622 (59.9)

Of the 1,037 mothers examined in this study, 16% were below 20 years of age, and 24% were 30 years or older. More than one-third (35%) of women were primigravida, 13% had no formal education, and 16.5% had a BMI <18.5. A relatively large proportion (44%) of women who came for the physical examination were in the two highest asset quintiles, and a similar percentage had received 8+ years of schooling. About 90.5% had a livebirth and 9.5% a stillbirth. About one in 20 mothers (5.5%) who delivered a live-born baby had experienced an early neonatal death. Over one-third of women (35.4%) had a caesarean section for their delivery. Among all mothers, 71% delivered in a basic or comprehensive emergency obstetric care (EmOC) facility in Matlab or Chandpur, and the rest 29% delivered at home.

Group-wise, about 54% and 64% of women in the categories of complicated births and caesarean sections without any maternal indication respectively had more than 8 years of schooling; this level was 35% in both the groups with a perinatal death or normal uncomplicated birth; 45% and 40% of women in the categories of complicated births and of caesarean sections without any maternal indication belonged to the richest wealth quintile; the corresponding figures were nearly half (23% and 18%) in the perinatal death and uncomplicated birth groups.

Table 3 describes the occurrence of various postpartum maternal morbidities/disabilities diagnosed at 6-9 weeks after delivery in sampled mothers by category of delivery. Upon clinical diagnosis among sample mothers, presence of at least one moderate to severe postpartum morbidity ranged from 27.6% in women having a caesarean section to 48.7% in those with perinatal death. About 42% of mothers with a normal uncomplicated birth also suffered from further physical consequences of pregnancy and delivery.

Of the different postpartum morbidities/disabilities, genital infection was the most common in all delivery categories, ranging from 14% in mothers having caesarean section without maternal indications to 27% in those with perinatal deaths. Urinary tract infection was higher in mothers with normal uncomplicated births (14.9%) and those with acute obstetric complications (12.0%) compared to below 10% in the other two groups. Perineal tears were relatively high among mothers with either normal uncomplicated births (18.4%) or perinatal deaths (15.7%); in the other two groups, the corresponding figure was low (1% or below).

Among longer-term morbidities, external haemorrhoids were the most common in all four categories (ranging from 5.98% to 10.37%). Hypertension was the second most common longer-term morbidity: About 8% of women in the acute obstetric complication group were diagnosed as hypertensive at 6-9 weeks postpartum. The corresponding figures varied from 6% to 3% in the other three groups. Levels of uterine prolapse were higher in women with an uncomplicated normal vaginal delivery (5.6%) and those with a perinatal mortality (4.5%) compared to the other two groups (with below 2%). Though severe anaemia was uncommon among all study subjects, overall 3.6% of women had moderate or severe anaemia postpartum, varying from 5.1% in the acute obstetric complication group to 1.6% in women who had caesarean section without maternal indications.

Table 4 provides crude and adjusted odds ratios (AOR) with 95% CIs for postpartum morbidities/disabilities among women with a complicated birth (severe or less-severe acute obstetric complication), a perinatal death, or a caesarean section without maternal complications compared to those with a normal uncomplicated birth.

Women with complicated births were more likely to suffer from hypertension (AOR=3.44; CI=1.12-10.36), haemorrhoids (AOR=1.73; CI=1.11-3.09) and moderate/severe anaemia (AOR=7.11; CI=2.03-4.88) compared to those with uncomplicated births. These same women, however, were less likely to have perineal tears (AOR=0.05; CI=0.02-0.14) or uterine prolapse (AOR=0.22; CI=0.06-0.76). Women with a perinatal death were more likely to have genital infections than those with an uncomplicated birth (AOR=1.92; CI=1.18-3.14) but less likely to have genital prolapse (AOR=0.48; CI=0.23-0.96) or urinary tract infections (AOR=0.40; CI=0.20-0.79). Urinary tract infections (AOR=0.31; CI=0.13-0.72) and genital prolapse (AOR=0.25; CI=0.10-0.59) were found significantly lower in mothers who had caesarean section than those who had uncomplicated deliveries.

Variations in the incidence of postpartum morbidities/disabilities for selected sociodemographic and reproductive characteristics are shown in Table 5. Older women (those equal to 30 years or older) were prone to have more incontinence (about 3% vs nearly 0% in younger women), be more hypertensive (9% compared to 4.5% and 1% in the women aged 20-29 and <20 years respectively), and suffered more prolapse (about 22% versus 10% and 2% women diagnosed with a prolapse in the 20-29 and <20 years age-groups respectively). Similarly, women with more than 4 children suffered more incontinence (9% compared to 0% in the women with fewer children) and prolapse (24% versus 16% and 4% in women with parity 2-4 and 1 respectively).

**Table 3.** Levels of postpartum morbidities and disabilities at 9 weeks postpartum by type of delivery

Type of maternal morbidity/disability	Complicated births/acute obstetric complication (n=274) No. (%)	CS without maternal indication (n=125) No. (%)	Perinatal death (n=156) No. (%)	Normal uncomplicated birth (n=482) No. (%)	Total (n=1,037) No. (%)	p value for chi-square
Postpartum morbidities						
Breast problem	0 (0.0)	1 (0.8)	1 (0.64)	4 (0.85)	6 (0.6)	0.69
Perineal tear	4 (1.48)	0 (0.0)	24 (15.7)	86 (18.38)	114 (11.2)	<0.001
Third-degree	1 (0.37)	0 (0.0)	0 (0.0)	3 (0.64)	4 (0.4)	-
Second-degree	0 (0.0)	0 (0.0)	5 (3.27)	6 (1.28)	11 (1.1)	-
First-degree (mild tear)	3 (1.11)	0 (0.0)	19 (12.42)	77 (16.45)	99 (9.8)	-
Urinary tract infection	33 (12.04)	8 (6.4)	14 (8.97)	72 (14.94)	127 (12.3)	0.06
Genital infection (clinical diagnosis)	53 (19.62)	17 (13.71)	41 (26.80)	78 (16.67)	189 (19.0)	0.04
Longer-term morbidity/disability						
Urinary incontinence	3 (1.11)	0 (0.0)	3 (1.96)	4 (0.85)	10 (1.0)	0.48
Uterine prolapse	2 (0.74)	2 (1.60)	7 (4.5)	27 (5.60)	38 (3.5)	<0.001
Hypertension	22 (8.02)	4 (3.2)	9 (5.77)	18 (3.73)	53 (5.1)	0.09
External haemorrhoids	28 (10.37)	8 (6.45)	15 (9.80)	28 (5.98)	79 (7.8)	<0.01
Anaemia						
Severe anaemia (Hb <7 g/dL)	1 (0.4)	0 (0.0)	0 (0.0)	1 (0.4)	2 (0.2)	-
Moderate (Hb 7-9 g/dL)	13 (4.74)	2 (1.60)	7 (4.64)	13 (2.7)	35 (3.4)	-
Mild (Hb >9-11 g/dL)	73 (26.83)	30 (24.0)	40 (26.49)	147 (30.69)	290 (28.3)	-
At least one postpartum morbidity/disability (excluding mild anaemia, first-degree tear)	126 (45.9)	34 (27.6)	76 (48.7)	203 (42.1)	439 (42.0)	-

CS=Caesarean section

**Table 4.** Odds ratios of postpartum morbidities/disabilities by category of delivery compared to those with normal uncomplicated delivery*

Morbidities/disabilities	Complicated birth/uncomplicated birth		Perinatal death/uncomplicated birth		C-section without maternal indication/uncomplicated birth	
	Crude OR (95% CI)	Adjusted OR (95% CI)	Crude OR (95% CI)	Adjusted OR (95% CI)	Crude OR (95% CI)	Adjusted OR (95% CI)
Breast problem	-	-	0.77 (0.08-6.94)	0.17 (0.006-4.93)	0.96 (0.10-8.6)	0.04 (0.01-1.48)
Urinary incontinence	1.6 (0.4-6.51)	-	2.32 (0.51-10.48)	3.69 (0.31-4.04)	-	1.11 (0.11-10.7)
Hypertension	2.28 (1.21-4.28)	3.44 (1.14-10.36)	1.57 (0.69-3.58)	1.46 (0.56-3.80)	0.85 (0.28-2.56)	0.52 (0.13-2.09)
External haemorrhoids	1.80 (1.05-3.09)	1.73 (1.01-3.09)	1.70 (0.88-3.29)	1.95 (0.92-4.13)	1.08 (0.48-2.44)	1.15 (0.43-3.08)
Perineal tear	0.06 (0.02-0.16)	0.05 (0.02-0.14)	0.79 (0.49-1.27)	1.00 (0.59-1.69)	0.03 (0.00-0.22)	0.04 (0.00-0.35)
Genital prolapse	0.19 (0.06-0.63)	0.22 (0.06-0.76)	0.45 (0.24-0.84)	0.48 (0.23-0.96)	0.25 (0.10-0.59)	0.34 (0.13-0.89)	
UTI	0.74 (0.47-1.14)	0.86 (0.36-2.03)	0.56 (0.30-1.00)	0.40 (0.20-0.79)	0.38 (0.18-0.83)	0.31 (0.13-0.72)	
Genital infection (clinical diagnosis)	1.16 (0.79-1.70)	1.74 (0.83-3.63)	1.83 (1.18-2.82)	1.92 (1.18-3.14)	0.79 (0.45-1.39)	0.73 (0.37-1.45)
Moderate/severe anaemia	2.21 (1.03-4.74)	7.11 (2.03-4.88)	1.61 (0.52-4.91)	0.56 (0.30-1.02)	0.61 (0.13-2.80)	1.25 (0.19-7.97)

*Adjusted for age, parity, SES, and education; CS=Caesarean section; CI=Confidence interval; SES=Socioeconomic status; UTI=Urinary tract infection

About 7% and 17% women who did not have any formal education were diagnosed as having anaemia and prolapse respectively; the corresponding figures were 2% and 8% in women with more than 8 years of schooling.

The women with perineal tears and prolapse nearly doubled (15% and 14%) in the poorest quintile in comparison with their counterparts in the richest quintile (8% with either morbidity). Twenty-one percent women who delivered at home developed perineal tears three times more than women who delivered at facility (7%). Similarly, more women with delivery at home suffered from prolapse than those who delivered at facility (16% versus 10%).

A high BMI was associated with hypertension and haemorrhoids. About 13% and 14% of women with BMI >25 were diagnosed with hypertension or haemorrhoids compared to 4% and 7% women respectively with BMI in the normal range (BMI=18.5-25).

The association of clinically-diagnosed postpartum morbidities/disabilities with sociodemographic characteristics, mode and place of delivery, nutritional status and outcomes of the baby are described in Table 6. As with the previous analysis, older women (≥30 years of age) were more likely to have hypertension, haemorrhoids, prolapse, and perineal tears compared to those who were younger (<20 years of age). Women with higher parity (4+) were more likely to have a prolapse than the primipara. Women from richer households were more likely to be diagnosed with haemorrhoids than those from the poor households. Overweight women (BMI >25) were more likely to suffer from hypertension than those who were underweight. The women who delivered at home had perineal tears 3.5 times more often than those with a delivery in hospital. Prolapse was higher in women with vaginal deliveries compared to those with a caesarean section. Women without any formal education were more likely to suffer from anaemia than those who had at least 8 years of schooling.

**Table 5.** Variations in occurrence of postpartum morbidities/disabilities by selected sociodemographic and reproductive characteristics of women

Sociodemographic characteristics	Postpartum morbidities/disabilities								
	Breast problem n/N (%)	Incontinence n/N (%)	Hypertension n/N (%)	Haemorrhoids n/N (%)	Perineal tear n/N (%)	Prolapse n/N (%)	UTI n/N (%)	Genital infection n/N (%)	Anaemia n/N (%)
Age (years)									
<20	0/165 (0.00)	0/164 (0.00)	2/165 (1.21)	5/164 (3.05)	11/164 (6.71)	4/164 (2.44)	20/164 (12.20)	35/164 (21.34)	7/163 (4.29)
20-29	4/615 (0.65)	2/601 (0.33)	28/615 (4.55)	48/601 (7.99)	67/601 (11.15)	59/601 (9.82)	62/601 (10.32)	101/601 (16.81)	19/609 (3.12)
30+	2/255 (0.78)	8/248 (3.23)	23/255 (9.02)	26/248 (10.48)	36/248 (14.52)	55/248 (22.18)	40/248 (16.13)	53/248 (21.37)	11/253 (4.35)
p value	0.54	<0.001	<0.01	0.02	0.04	<0.001	0.06	0.188	0.59
Parity
1	3/362 (0.83)	1/356 (0.28)	14/362 (3.87)	20/356 (5.62)	33/356 (9.27)	13/356 (3.65)	46/356 (12.92)	72/356 (20.22)	15/357 (4.20)
2-4	3/515 (0.58)	5/504 (0.99)	27/515 (5.24)	44/504 (8.73)	66/504 (13.10)	85/504 (16.87)	52/504 (10.32)	90/504 (17.86)	16/511 (3.13)
4+	0/36 (0.00)	3/33 (9.09)	3/36 (8.33)	3/33 (9.09)	8/33 (24.24)	8/33 (24.24)	8/33 (24.24)	8/33 (24.24)	2/35 (5.71)
p value	0.80	<0.001	0.39	0.21	0.02	<0.001	0.04	0.50	0.57
Maternal education (years of schooling)
No education	2/136 (1.47)	4/132 (3.03)	6/136 (4.41)	9/132 (6.82)	18/132 (13.64)	23/132 (17.42)	21/132 (15.91)	22/132 (16.67)	9/134 (6.72)
<8	0/332 (0.00)	1/323 (0.31)	14/332 (4.22)	26/323 (8.05)	42/323 (13.00)	44/323 (13.62)	35/323 (10.84)	58/323 (17.96)	12/329 (3.65)
8+	4/453 (0.88)	3/447 (0.67)	20/453 (4.42)	33/447 (7.38)	44/447 (9.84)	35/447 (7.83)	48/447 (10.74)	89/447 (19.91)	11/448 (2.46)
p value	0.13	0.015	0.99	0.88	0.28	0.002	0.23	0.63	0.06
Socioeconomic status									
Poor	2/317 (0.63)	3/305 (0.98)	16/317 (5.05)	14/305 (4.59)	45/305 (14.75)	42/305 (13.77)	39/305 (12.79)	47/305 (15.41)	14/315 (4.44)
Middle	0/175 (0.00)	2/172 (1.16)	11/175 (6.29)	13/172 (7.56)	21/172 (12.21)	30/172 (17.44)	23/172 (13.37)	37/172 (21.51)	5/173 (2.89)
Rich	3/455 (0.66)	5/448 (1.12)	24/455 (5.27)	41/448 (9.15)	38/448 (8.48)	38/448 (8.48)	46/448 (10.27)	86/448 (19.20)	14/450 (3.11)
p value	0.56	0.97	0.83	0.06	0.02	0.004	0.42	0.21	0.54
Place of delivery									
Hospital	4/735 (0.54)	5/725 (0.69)	42/735 (5.71)	61/725 (8.41)	51/725 (7.03)	72/725 (9.93)	88/725 (12.14)	133/725 (18.34)	23/727 (3.16)
Home	2/302 (0.66)	5/290 (1.72)	11/302 (3.64)	18/290 (6.21)	63/290 (21.72)	46/290 (15.86)	34/290 (11.72)	56/290 (19.31)	14/300 (4.67)
p value	0.82	0.13	0.16	0.23	<0.001	0.008	0.85	0.72	0.24
BMI (kg/m^2^)									
<18.5	3/170 (1.76)	0/164 (0.00)	5/170 (2.94)	9/164 (5.49)	22/164 (13.41)	19/164 (11.59)	17/164 (10.37)	31/164 (18.90)	7/168 (4.17)
18.5-25	3/743 (0.42)	5/731 (0.68)	33/743 (4.42)	53/731 (7.25)	80/731 (10.94)	88/731 (12.04)	91/731 (12.45)	137/731 (18.74)	28/741 (3.78)
>25	0/120 (0.00)	5/120 (4.17)	15/120 (12.50)	17/120 (14.17)	12/120 (10.00)	11/120 (9.17)	14/120 (11.67)	21/120 (17.50)	2/118 (1.69)
p value	0.07	0.001	<0.000	0.016	0.59	0.66	0.75	0.94	0.48
Mode of delivery
Vaginal	5/670 (0.75)	7/654 (1.07)	33/670 (5.03)	44/654 (6.73)	114/654 (17.43)	101/654 (15.44)	92/670 (13.61)	127/654 (19.42)	26/662 (3.89)
Caesarean section	1/367 (0.27)	3/361 (0.83)	20/367 (5.45)	35/361 (9.70)	0/361 (0.00)	17/361 (4.70)	35/367 (9.54)	62/361 (17.17)	11/365 (3.01)
p value	0.33	0.71	0.71	0.09	-	<0.001	0.04	0.37	0.22

UTI=Urinary tract infection

**Table 6.** Association of postpartum morbidities/disabilities (Crude and Adjusted OR with 95% CI) with sociodemographic and reproductive characteristics

Sociodemographic characteristics	OR/AOR	Hypertension	Genital infection	Haemorrhoids	Prolapse	Perineal tear	Anaemia	UTI	Breast problem	Incontinence
Age (years) <20 (reference)										
20-29	Crude OR (95% CI)	2.38 (0.91-16.41)	0.77 (0.48-1.14)	2.76 (1.08-7.05)	4.35 (1.55-12.17)	1.74 (0.89-3,38)	0.71 (0.29-1.73)	0.82 (0.48-1.41)	-	-
	Adjusted OR (95% CI)	3.66 (0.82-16.24)	0.77 (0.48-1.24)	2.67 (1.07-7.09)	3.14 (1.07-9.20)	1.81 (0.88-3.37)	0.82 (0.31-2.14)	0.96 (0.53-1.720	-	-
30+	Crude OR (95% CI)	8.07 (1.87-34.74)	1.0 (0.61-1.62)	3.72 (1.39-9.90)	11.39 (4.04-32.13)	2.36 (1.16-4.78)	1.01 (0.38-2.66)	1.38 (0.77-2.46)	-	-
	Adjusted OR (95% CI)	8.04 (1.65-39.16)	1.06 (0.59-1.92)	3.33 (1.13-9.83)	6.34 (2.05-19.6)	2.35 (1.01-5.51)	1.09 (0.32-3.67)	1.67 (0.81-3.42)	-	-
Parity 1 (reference)										
2-4	Crude OR (95% CI)	1.37 (0.71-2.66)	0.85 (0.60-1.21)	1.60 (0.92-2.77)	5.35 (2.93-9.76)	1.47 (0.94-2.29)	0.73 (0.35-1.51)	0.77 (0.50-1.18)	0.70 (0.14-3.49)	3.55 (0.41-30.57)
	Adjusted OR (95% CI)	0.74 (0.34-1.61)	0.90 (0.59-1.36)	1.35 (0.73-2.51)	3.12 (1.62-5.98)	0.91 (0.53-1.55)	0.58 (0.24-1.40)	0.50 (0.30-0.83)	0.22 (0.02-1.83)	1.93 (0.15-23.75)
4+	Crude OR (95% CI)	2.24 (0.61-8.26)	1.26 (0.54-2.91)	1.68 (0.47-5.98)	8.44 (3.20-22.26)	3.13 (1.30-7.49)	1.38 (0.30-6.30)	2.15 (0.91-5.06)	-	35.5 (3.58-351.8)
	Adjusted OR (95% CI)	0.73 (0.16-3.29)	1.23 (0.47-3.18)	1.21 (0.29-4.98)	2.97 (1.02-8.69)	1.90 (0.64-5.62)	0.73 (0.12-4.32)	0.93 (0.34-2.54)	-	14.01 (0.62-314.70)
Maternal education No education (reference)									
<8 years	Crude OR (95% CI)	0.95 (0.35-2.53)	1.09 (0.63-1.87)	1.19 (0.54-2.62)	0.74 (0.43-1.29)	0.96 (0.52-1.71)	0.52 (0.21-1.27)	0.64 (0.35-1.15)	-	0.09 (0.01-0.89)
	Adjusted OR (95% CI)	1.21 (0.44-3.32)	1.09 (0.62-1.92)	1.24 (0.54-2.82)	1.11 (0.61-2.03)	1.33 (0.69-2.53)	0.50 (0.19-1.30)	0.62 (0.33-1.16)	-	0.11 (0.009-1.37)
8+	Crude OR (95% CI)	1.00 (0.39-2.54)	1.24 (0.74-2.07)	1.08 (0.50-2.33)	0.40 (0.22-0.70)	0.69 (0.38-1.24)	0.34 (0.14-0.86)	0.63 (0.36-1.10)	0.59 (0.10-3.29)	0.21 (0.04-0.97)
Adjusted OR (95% CI)	1.18 (0.40-3.45)	1.30 (0.72-2.34)	0.80 (0.33-1.90)	0.89 (0.45-1.76)	1.91 (0.94-5.62)	0.35 (0.12-1.04)	0.75 (0.39-1.46)	1.22 (0.08-17.31)	0.50 (0.06-4.23)
SES Poor (reference)										
Middle	Crude OR (95% CI)	1.26 (0.57-2.78)	1.50 (0.93-2.42)	1.69 (0.77-3.70)	1.32 (0.79-2.20)	0.80 (0.46-1.40)	0.63 (0.22-1.80)	1.05 (0.60-1.83)	-	1.18 (0.19-7.15)
Adjusted OR (95% CI)	1.26 (0.57-2.78)	1.45 (0.88-2.38)	1.81 (0.81-4.04)	1.41 (0.81-2.47)	0.79 (0.43-1.44)	0.77 (0.26-2.25)	0.99 (0.55-1.77)	-	2.84 (0.36-22.26)
Rich	Crude OR (95% CI)	1.04 (0.54-2.00)	1.30 (0.88-1.92)	2.09 (1.12-3.91)	0.58 (0.36-0.92)	0.53 (0.33-0.84)	0.69 (0.32-1.46)	0.78 (0.49-1.22)	1.0 (90.17-6.29)	1.13 (0.19-7.15)
	Adjusted OR (95% CI)	1.08 (0.50-2.30)	1.30 (0.83-2.04)	2.30 (1.15-4.59)	0.88 (0.50-1.53)	0.71 (0.40-1.24)	1.10 (0.45-2.65)	0.82 (0.48-1.40)	0.78 (0.05-12.17)	1.40 (0.20-9.8)
Place of delivery Hospital (reference)										
Home	Crude OR (95% CI)	0.62 (0.31-1.22)	1.06 (0.75-1.50)	0.72 (0.41-1.24)	1.70 (1.14-2.54)	3.66 (2.46-5.46)	1.49 (0.76-2.95)	0.96 (0.63-1.46)	1.21 (0.22-6.68)	2.52 (0.72-8.79)
	Adjusted OR (95% CI)	0.52 (0.24-1.12)	1.13 (0.75-1.69)	0.90 (0.47-1.71)	0.90 (0.57-1.41)	3.53 (2.32-5.37)	1.35 (0.60-3.05)	0.68 (0.42-1.10)	0.78 (0.10-5.77)	7.3 (0.94-57.73
BMI <18.5 (reference)										
18.5-25	Crude OR (95% CI)	1.52 (0.58-3.96)	0.98 (0.64-1.52)	1.34 (0.65-2.78)	1.04 (0.61-1.77)	0.79 (0.47-1.31)	0.90 (0.38-2.10)	1.22 (0.71-2.12)	0.22 (0.04-1.12)	-
	Adjusted OR (95% CI)	1.30 (0.48-3.51)	0.93 (0.60-1.46)	1.07 (0.50-2.26)	0.90 (0.51-1.60)	0.82 (0.47-1.42)	0.95 (0.40-2.29)	1.31 (0.74-2.32)	0.11 (0.01-0.69)	-
>25	Crude OR (95% CI)	4.71 (1.66-13.15)	0.91 (0.49-1.67)	2.48 (1.22-6.62)	0.77 (0.35-1.68)	0.71 (0.33-1.51)	0.399 (0.08-1.94)	1.149 (0.53-2.41)	-	-
	Adjusted OR (95% CI)	3.75 (1.23-11.37)	0.85 (0.45-1.63)	1.87 (0.76-4.57)	0.74 (0.31-1.73)	1.15 (0.50-2.65)	0.45 (0.08-2.37)	1.37 (0.62-3.04)	-	-
Mode of delivery Vaginal (reference)										
CS	Crude OR (95% CI)	1.12 (0.62-1.96)	0.86 (0.61-1.20)	1.48 (0.93-2.36)	0.27 (0.15-0.46)	-	0.76 (0.37-1.55)	0.66 (0.44-0.99)	0.36 (0.04-3.12)	0.77 (0.19-3.01)
	Adjusted OR (95% CI)	0.86 (0.43-1.72)	0.95 (0.63-1.44)	1.34 (0.80-2.24)	0.29 (0.16-0.54)	-	1.05 (0.43-2.57)	0.47 (0.29-0.77)	0.42 (0.10-4.58)	0.97 (0.19-4.79)
Pregnancy outcome Live baby (reference)										
Perinatal death	Crude OR (95% CI)	1.16 (0.55-2.43)	1.76 (1.18-2.63)	1.35 (0.75-2.44)	0.66 (0.36-1.22)	1.59 (0.98-2.59)	1.37 (0.59-3.17)	0.58 (0.31-1.08)	1.13 (0.13-9.74)	2.44 (0.62-9.55)
	Adjusted OR (95% CI)	1.01 (0.46-2.25)	1.83 (1.21-2.78)	1.42 (0.76-2.64)	0.55 (0.28-1.06)	1.79 (1.07-2.98)	1.21 (0.49-2.99)	0.58 (0.20-0.76)	1.74 (0.13-22.89)	3.23 (0.61-17.14)

*Adjusted for age, parity, maternal education, SES, and place of delivery; CS=Caesarean section; SES=Socioeconomic status; UTI=Urinary tract infection

## DISCUSSION

The icddr,b intervention area in Matlab provides a unique opportunity to follow women through the postpartum period and beyond for physical consequences, including longer-term postpartum morbidities/disabilities. Among the sample mothers, nearly half (42%) of all women in our study cohort suffered from at least one morbidity/disability at 6-9 weeks after delivery as diagnosed by a physician. Although most of these morbidities or disabilities were mild, there was a moderately-high rate of genital infection (19%) and of urinary tract infections (12.3%). Genital infection was higher in women with a perinatal death, suggesting unhygienic delivery-care or a pre-existing infection that could lead to the perinatal death.

One possible reason for the mildness of most problems is that the women could have received care for many problems at the time of delivery. For example, 11 women in the complicated delivery group, who suffered a severe haemorrhage (21), had a blood transfusion, contributing to the low rates of moderate and severe anaemia were found in the postpartum period for that group. Caesarean-section delivery emerged as a comparably strong protective factor for prolapse, perineal tears, incontinence, and urinary tract infection as expected, although such surgery is known to have other adverse effects (e.g. adhesion of internal organs and lower back-pain). We did not specifically examine women for the consequences of caesarean-section delivery.

Even when delivery was ‘normal and uncomplicated’, nearly half of the women suffered further physical consequences of pregnancy and delivery: perineal tears, prolapse, and increased risks of infections. Fifty-two percent of these women delivered at home, often without any trained birth attendant. The perineal tears, prolapse, and infections may be a consequence of problems suffered in the course of labour and delivery at home or caused by improper intrapartum care. Women who experienced dystocia and were hospitalized largely averted these same consequences, probably due to caesarean section: 78% of women with complicated deliveries had a caesarean section due to dystocia.

Other studies with physician-diagnosed morbidities have typically reported high rates of postpartum problems. Most of these studies report population-based rates, not rates linked to prior obstetric complications or delivery management, and so are not comparable with findings of our study. In Bangladesh, a community-based cohort study conducted in an urban slum area in Dhaka reported that 75% women were suffering from postpartum morbidities (23). Using clinical diagnosis by a physician, they reported 10% uterine prolapse, 17% perineal tear, 7% severe anaemia, and 11% hypertension. Another community-based cohort study in a district of South India reported a high burden of clinically-diagnosed gynaecological morbidity of women within one year of childbirth and showed that approximately one-fourth of the women had clinical evidence of pelvic inflammatory disease or fistula after physical examination; sexually transmitted diseases contributed 10% to the overall burden. This same study also reported that 17% of study subjects had severe anaemia, and 12% had severe chronic energy deficiency (24).

More similar to our study linking postpartum morbidities to complications and management at delivery is a recent study in Burkina Faso that explored the physical consequences of delivery among women with severe intrapartum obstetric complications and normal uncomplicated deliveries. They found little significant differences between groups in terms of urinary tract infections, hypertension, haemorrhoids, and prolapse. The study only found a notably-high incidence of anaemia in the complicated birth group when compared with the uncomplicated birth group (1).

In our study, among women who suffered complicated births/acute obstetric complications around the time of delivery, hypertension, haemorrhoids, and severe to moderate anaemia were found significantly higher than among those with uncomplicated births. Hypertension and haemorrhoids (8.0% and 10.4% among women with complicated births) could develop during pregnancy and delivery respectively and persist as postpartum consequences. A perineal tear is likely to be a direct consequence of the last vaginal delivery. Yet, the health of the women before their last delivery was not checked; it is difficult to say that prolapse was a consequence of the last normal vaginal delivery. Further methodological research is needed to better understand the different consequences as related to a specific obstetric complication.

The socioeconomic and demographic risk factors also contributed to those suffering from hypertension, haemorrhoids, perineal tears, prolapse, or anaemia. Hypertension was highly associated with increased age and obesity. Haemorrhoids were found higher in the rich and older women. Prolapse was more common among the older, multiparity women and those who delivered vaginally. Tears were associated with older mothers and those with delivery at home. Although the perineal tears were mostly mild and healed during the time of postpartum examination (except for the few second- and third-degree tears: 1.5%), women who had delivery at home, attended by an unskilled care provider, might be a reason for more tears. There was a high incidence of anaemia among illiterate mothers. The above findings suggest that socioeconomic status as a risk factor is a strong predictor for some long-term postpartum physical morbidities and disabilities, e.g. hypertension, prolapse, and haemorrhoids.

### Limitations

The study had several limitations. While compliance of women for interview and genital examination by a physician were appreciable, still 10.8 % refused to participate or were lost to follow-up. These women were not different from the examined category in terms of background characteristics as shown in Table 1. The study was able to examine 89.2% of women recruited; this high coverage is most likely due to the long existence of a safe motherhood programme in Matlab, the familiarity of the mothers in the icddr,b study area with the midwives and doctors, and the knowledge that free treatment was provided for any diagnosed morbidity.

As we recruited women with obstetric complications at birth from among those with delivery at hospital, there was a higher proportion of women in the sample, who were richer/more educated as they attended facilities for delivery more than their poorer counterparts. These same women are likely to have been better-nourished and healthier at delivery. Unfortunately, from the sample with a normal birth at home, we lacked medical information about their condition at home before and during the delivery. Even so, we reasoned that, if any woman had a severe complication and survived, she would likely have accessed one of the local hospitals for care.

This lack of data on prior health status at birth is a serious limitation of the study. Some of the postpartum morbidities, e.g. prolapse, haemorrhoids, hypertension, and anaemia, could have presented before the last delivery. For a certain proportion of women, prolapse diagnosed at 6-9 weeks postpartum most likely pre-existed. Anaemia could also be associated with other causes, such as undernutrition, and may not have been caused by the last delivery, although pregnancy and delivery would most likely exacerbate any pre-existing anaemia.

Urinary incontinence was difficult to measure using the Cough Reflex Test, as the test was carried out at only one time-point. Physical examination of gynaecological morbidities obviously has its limitations.

The sample does not represent population levels of postpartum morbidities among women in rural Matlab. It was rather selected to learn about the consequences of acute maternal morbidities, service provision (normal vaginal birth versus caesarean section), and of perinatal mortality among women within a short time after delivery.

### Conclusion and recommendations/programmatic implications

These data confirm the value of accessible medical support in the face of obstetric complications and for normal labour and highlight the potential benefits and importance of high-quality delivery and postpartum care. For example, while the complicated birth group proceeded to hospital for delivery, and thus, averted consequences, such as incontinence, tears, or prolapse, they did continue to suffer from hypertension, haemorrhoids, or anaemia in the postpartum period. There is a need for specific postpartum interventions to address these problems. Prolapse and haemorrhoids could be treated in the first-line referral hospitals following delivery while the women are still there, thus lowering the burden of these long-term postpartum sequelae. One-fourth of the sample mothers had mild anaemia during the time of postpartum examination. Effective programmes for health education and iron supplementation are important for preventing anaemia, which typically are not provided in the postpartum period (25). Women who have experienced perinatal death should certainly not be ignored in the postpartum period as these data demonstrate their high risk of genital infection.

Last but hardly the least, ensuring that skilled birth attendants (SBAs) assist in all deliveries is equally important to improve postpartum care. The SBAs can identify and ensure timely and appropriate management of obstetric complications or stabilize the woman and coordinate a referral to places which can manage the complications.

## ACKNOWLEDGEMENTS

This study was funded by the United States Agency for International Development (USAID), (Grant No. GHS-A00-0300019-00), and Department for International Development (DFID) (Grant No. D-00024) through a Research Programme Consortium to icddr,b; icddr,b acknowledges with gratitude the commitment of USAID and DFID to its research efforts. Dr. Chowdhury was partially supported by a training grant from the National Institutes of Health Fogarty International Center TW007587-04 to the Johns Hopkins University.
